# Predictive value of admission CO_2_ combining power combined with serum sodium for the prognosis in acute Stanford type A aortic dissection patients

**DOI:** 10.1038/s41598-022-27099-6

**Published:** 2023-01-19

**Authors:** Peng-fei Huang, Yun-jing Zhang, Xian-zhe Lou, Dong Ma, Yun-yan Wu, Yong-bo Zhao

**Affiliations:** 1 School of Public Health, North China University of Science and Technology, Tangshan, 063210 Hebei People’s Republic of China; 2 Department of Biochemistry and Molecular Biology, Key Laboratory of Neural and Vascular Biology, Ministry of Education, Hebei Medical University, Shijiazhuang, 050017 Hebei People’s Republic of China; 3 Cardiac Surgery Department, the Fourth Hospital of Hebei Medical University, Shijiazhuang, 050017 Hebei People’s Republic of China

**Keywords:** Prognostic markers, Cardiovascular diseases

## Abstract

Acute Stanford type A aortic dissection (ATAAD) with sudden onset and high mortality requiries a standard Bentall operation and a accurate prognosis in common, together with alteration of CO_2_ combining power (CO_2_CP) and serum sodium rase concern, hence, we evaluated the prognostic value of CO_2_CP combined with serum sodium in ATAAD patients. This retrospective study included 183 patients who underwent Bentall operation for ATAAD from 2015 to 2021 in the Fourth Hospital of Hebei Medical University, subsequently followed grouping by the levels of CO_2_CP and serum sodium. The study endpoint was 30-day all-cause mortality, and the prognostic value of CO_2_CP combined with serum sodium levels in ATAAD patients were evaluated with multivariate logistic regression method. The postoperative incidence of in-hospital death and adverse events in patients with ATAAD were 18% and 25.7%, respectively. Combination of CO_2_CP and serum sodium for predicting ATAAD death and adverse events presented a higher predictive value than each single indicator with ROC curve analysis (the AUC of CO_2_CP combined with serum sodium was 0.786, 95% CI 0.706–0.869*, P* < 0.001), along with CO_2_CP < 22.5 mmol/L + serum sodium > 138.5 mmol/L group had the worst prognostic. Multivariate regression analyse showed that CO_2_CP < 22.5 mmol/L combined with serum sodium > 138.5 mmol/L preferably predicted the prognosis of ATAAD (OR =6.073, 95% CI 2.557–14.425, *P* < 0.001). Consistently, the cumulative 30-day survival after surgery in ATAAD patients with the low CO_2_CP and high serum sodium simultaneously was the worst (log-rank *P* < 0.05). The combination of CO_2_CP and serum sodium increases the predictive value of prognosis, which is conducive to risk stratification of patients with ATAAD.

## Introduction

Acute Stanford type A aortic dissection (ATAAD) is a life-threatening cardiovascular disease with high mortality^1^, and the population-based incidence of ATAAD is stated between 5 and 12 cases/100,000 persons inhabitants/year^2,3^. Without the only effective surgery treatment, the mortalities at 48 h and 2 weeks from the onset are 50% and 80%, respectively^4^. ATAAD is also one of the four differential diagnoses of acute chest pain at the emergency department. The pathology department information showed, that only one in two cases of ATAAD was diagnosed^3^ and the rate of initial misdiagnosis was 16% in previous study^5^. The wide variety of symptoms, which are associated with acute aortic dissection, make it difficult to establish the correct diagnosis. Timely diagnosis is essential for successful management hat requires quick and accurate diagnosis as a delay in treatment carries a high mortality rate^6,7^. Variables associated with delayed diagnosis include female sex, the absence of typical historical features (abrupt-onset, chest, or back pain), or high-risk examination findings, including hypotension or pulse deficits^8^.

Although immediate surgical treatment has greatly reduced the mortality of ATAAD patients, the early postoperative mortality rate of ATAAD patients maintaints high with postoperative in-hospital mortality ranging from 7 to 27%^9,10^. Numerous mortality-related variables have been identified, such as lymphocyte to monocyte ratio, systolic blood pressure, N-terminal pro-brain natriuretic peptides, C-reactive protein, cardiac troponins, fibrinogen, etc.^11–16^; however, effective variables remained inadiquate, urging us to develop some novel predictors on the ATAAD prognosis in clinical practice.

In clinic, CO_2_ combing power (CO_2_CP) as a low cost and time-saving auxiliary diagnosis usually reflects the homeostasis of acidosis and alkalosis. Accumulating evidence shows the low CO_2_CP level linking with the worse prognosis of patients with olorectal cancer, acute kidney injury, acute ischaemic stroke^17–19^. Similarly, the latest reports also indicates the increased lactic acid as an independent risk factor for ATAAD complications, including postoperative acute kidney and lung injury^20,21^, thereby driving the application of acid–base balance index on prognosis. Significantly, Liao et al.^22^ reported that admission low serum level of bicarbonate (HCO_3_) can predict short-term and long-term mortality in patients with ATAAD. Consistantly, the association between decreased CO_2_CP level at admission and poor prognosis in patients undergoing Sun’s procedure for ATAAD was observed in our daily work^23^.

Abnormal serum sodium during hospitalization are risk factors for poor prognosis^24,25 ^. Subsequently, we also verified that admission serum sodium of acute aortic dissection (including Stanford type B) patients play a vital on the postoperative hospital mortality^26^, whereas the link and interaction of CO_2_CP and serum sodium levels affecting all-cause death and adverse events of patients with ATAAD in context of different pathological type and surgical treatment is supposed to elaborated comprehensively. In addition, it should be noted that the predictive value of a single serum biomarker for ATAAD patients is usually limited, and it is difficult to simultaneously consider the sensitivity and specificity of prediction.

Therefore, this study focus on facilitating the prognosis prediction and outcome improvement via exploring the effect of admission CO_2_CP combined with serum sodium levels on the postoperative prognostic after Bentall procedure in patients with ATAAD.

## Materials and methods

### Study design and participants

In this retrospective study, patients with ATAAD admitted to the Fourth Hospital of Hebei Medical University from 2015 to 2021 were enrolled and were radiological proven as type A aortic dissection by computed tomographic angiography (CTA) by the Stanford classification, following underwent Bentall procedure for ATAAD repair as well as had a disease course within 14 days with complete clinical information. Patients were excluded from this analysis if: (1) ill for more than 14 days, (2) previous aortic, (3) connective tissue diseases, pregnancy, traumatic dissection, or infection diseases, (4) patients without Bentall procedure, (5) severe preoperative complications (including serum creatinine at admission > 115 µmol/l, a preoperative stroke or unconscious on admission, respiratory insufficiency, and malignant tumor), (6) incomplete clinical data.

Collection and analysis of demographic and clinical data were approved by the ethics committee of the Fourth Hospital of Hebei Medical University (2021k7359). As patient data were anonymized, the ethics committee of the Fourth Hospital of Hebei Medical University waived the written informed consent. All procedures followed were in accordance with the revised Declaration of Helsinki.

### Clinical data

Clinical variables of enrolled patients with ATAAD was obtained through review of medical records, including gender, age, medical history (hypertension, diabetes, coronary artery disease, prior surgery, prior trauma, smoking and drinking), complications, vital signs on admission (including systolic blood pressure, diastolic blood pressure, heart rate, etc.) and laboratory testing data on admission (alanine transaminase, aspartate transaminase, creatine, urea nitrogen, serum glucose, white blood cell count, neutrophil count, serum sodium, CO_2_CP, anion gap, etc.) as well as the duration of in-hospital. The laboratory data were obtained from the patients’ first venous blood samples taken at admission. The outcome of postoperative in-hospital patients with ATAAD was gotten from medical records, including operation selection and complicated with hypoperfusion syndrome etc. Hypoperfusion syndrome was defined as the reduced blood flow to one or more organs, resulting in organ ischemia and dysfunction^1^. Quality control was carried out in data collection to ensure accuracy.

### Surgical techniques

Bentall procedure is a standard technique for complete aortic root replacement and all patients with ATAAD underwent a successful Bentall procedure within 24 h of admission. The surgical technique has been described in the literature^27^.

### Observation endpoints

30-day all-cause mortality during hospitalization was defined as the primary endpoint. 30-day adverse events were served as the secondary endpoint. Adverse events included postoperative acute kidney injury, postoperative hypoxemia, low cardiac output syndrome and arrhythmia.

### Statistical analysis

All continuous variables were normality tested by Shapiro–Wilk test. Data that was normally distributed were shown as mean ± SD, one-way ANOVA was applied between four groups. The measurement variables of non-normal distribution were represented by the median (P25, P75) with Kruskal–Wallis test. Categorical variables were presented as frequency (%), and Chi-squared test or Fisher's exact test was used for comparison between groups. Area under curve (AUC) and optimal cut-off value were computed by receiver operating characteristic (ROC) curve to evaluate the prediction value of admission CO_2_CP and serum sodium level in postoperative all-cause death and adverse events. Optimal cut-off value was determined as the point at which the Youden index (sensitivity + specificity − 1) was maximal. Based on CO_2_CP and serum sodium optimal cut-off value (22.5 mmol/L, 138.5 mmol/L, respectively), the patients were divided into four groups: group A (CO_2_CP ≥ 22.5 mmol/L and serum sodium>138.5 mmol/L); group B (CO_2_CP ≥ 22.5 mmol/L and serum sodium ≤ 138.5 mmol/L); group C (CO_2_CP < 22.5 mmol/L and serum sodium > 138.5 mmol/L); group D (CO_2_CP < 22.5 mmol/L and serum sodium ≤ 138.5 mmol/L). Univariate and multivariate logistic regression analysis were performed to examine the influencing factors of postoperative prognostic and to calculate OR and 95% CI. Multivariable analysis was adjusted for potential confounders: age, gender and a statistically significant association at *P* < 0.05 in univariate regression analysis. The cumulative survival curves in different groups were presented as Kaplan–Meier curves and compared by log-rank test. All statistical analyses were performed using SPSS 25.0 and GraphPad Prism 8.0, and a value of *P* < 0.05 was considered statistically significant.

### Ethics approval and consent to participate

All clinical data related to the study were obtained after approved by the ethics committee of the Fourth Hospital of Hebei Medical University (2021k7359). All procedures followed in accordance with the revised Declaration of Helsinki.

## Results

### The ROC curve analysis of the CO_2_CP and serum sodium to predict prognosis in patients with ATAAD

**Figure 1:**
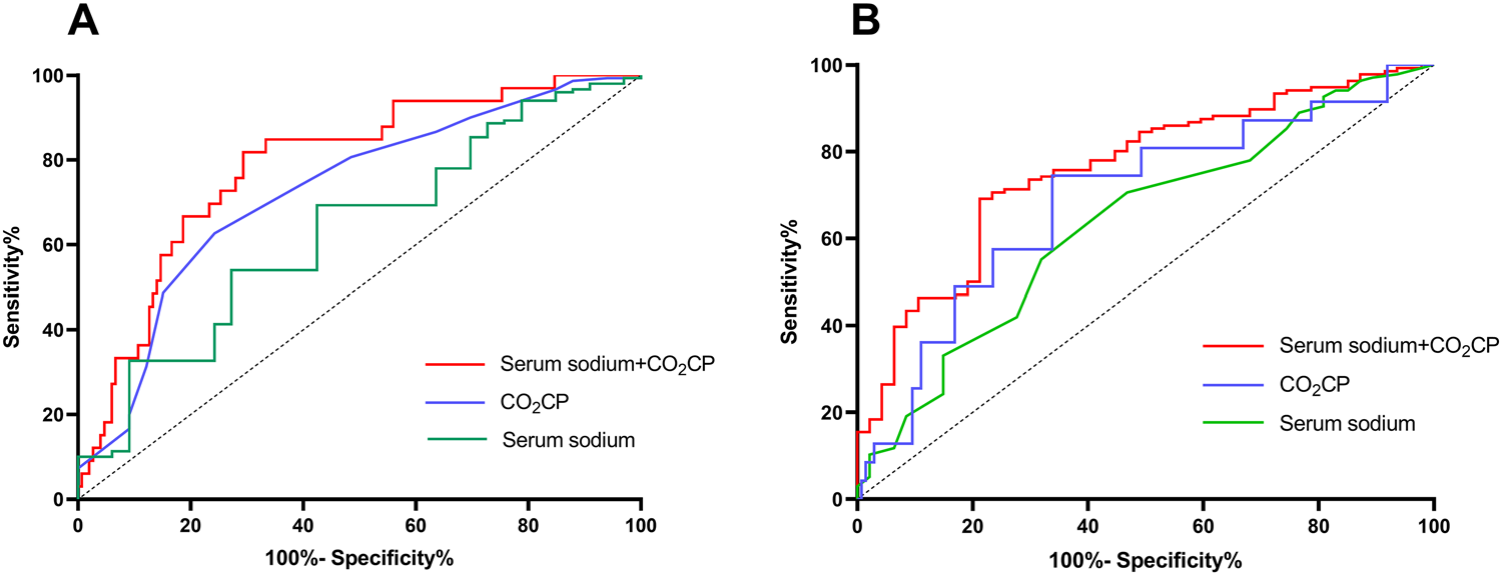
ROC curves comparing CO_2_CP, serum sodium and its combination for prediction. (**A**) All-cause death; (**B**) adverse events.

183 patients were enrolled in this study. The postoperative incidence of in-hospital death and adverse events in patients with ATAAD were 18% (n = 33) and 25.7% (n = 47), respectively. Fifteen patients died of aortic root rupture, three died of postoperative infection and six patients died of postoperative acute kidney injury. The predicting efficiency of indicators were shown in Fig. 1A,B and Tables 1 and 2. The results of ROC curve analysis showed a higher predictive value for all-cause death and adverse events in CO_2_CP + serum sodium combination than each single indicator, seperately.

**Table 1:** ROC curves analysis for all-cause death in ATAAD.

Variables	AUC	95%CI low	95%CI high	Sensitivity (%)	Specificity (%)	Cut-off value	*P*
CO_2_CP	0.727	0.629	0.824	62.7	75.8	22.5 mmol/L	< 0.001
Serum sodium	0.667	0.567	0.760	72.7	54.0	138.5 mmol/L	0.003
CO_2_CP + serum sodium	0.786	0.706	0.869	84.8	66.0	–	< 0.001

**Table 2:** ROC curves analysis for adverse events in ATAAD.

Variables	AUC	95%CI low	95%ci high	Sensitivity (%)	Specificity (%)	Cut-off value	*P*
CO_2_CP	0.727	0.641	0.812	66.2	74.5	22.5 mmol/L	< 0.001
Serum sodium	0.637	0.545	0.729	68.1	55.1	138.5 mmol/L	0.005
CO_2_CP + serum sodium	0.762	0.686	0.857	78.7	69.1	–	< 0.001

### Preoperative baseline characteristics and postoperative early outcomes of patients with ATAAD

The average age of the participants was 51.3 ± 11.6 (22–82) years old, and about 67.2% (n = 123) of them were male. According to the cut-off value of CO_2_CP and serum sodium, all patients were divided into four groups (A–D). The demographic and clinical characters of patients with ATAAD were described as in Table 3. Compared with patients in group A, B and D, patients in group C had a higher serum chloride levels, and a higher anion gap (all *P* < 0.01).

**Table 3:** Demographic characteristics and clinical data of patients (n = 183).

Variables	Group A (n = 47)	Group B (n = 55)	Group C (n = 46)	Group D (n = 35)	*P*
Age (years)	52.7 ± 9.2	50.8 ± 12.5	53.7 ± 12.0	48.9 ± 12.2	0.241
Male (n, %)	33, 70.2	37, 67.3	29, 63.0	24, 68.6	0.900
Hypertension (n, %)	1, 87.2	37, 67.3	34, 73.9	23, 65.7	0.079
Diabetes (n, %)	1, 2.1	0, 0	2, 4.3	0. 0	0.300
CAD (n, %)	1, 2.1	7, 12.7	3 , 6.5	0, 0	0.051
Smoking (n, %)	24, 51.1	26, 47.3	21, 45.7	20, 57.1	0.743
Drinking (n, %)	23, 48.9	30, 54.5	25, 54.3	23, 62.8	0.123
History of trauma (n, %)	5, 10.6	2,3.6	2, 4.3	0, 0	0.150
Surgical history (n, %)	16, 34.0	13, 23.6	11, 23.9	14, 40	0.270
Heart rate (beats/min)	76.6 ± 163	78.8 ± 18	81.1 ± 14.4	77.0 ± 14.4	0.523
SBP (mmHg)	130.5 ± 25.6	138.5 ± 24.3	129.3 ± 31.6	139.0.2 ± 24.8	0.148
DBP (mmHg)	73.2 ± 16.6	81.1 ± 14.9	73.3 ± 18.5	78.6 ± 18.8	0.052
Length of hospital (day)	17.0 (11.0, 24.0)	18.5 (14.0, 35.0)	18.0 (2.5, 24.0)	17.0 (11.0, 22.0)	0.539
ALT (U/L)	19.0 (14.0, 30.3)	23.6 (16.9, 29.7)	23.0 (16.3, 38.6)	21.0 (15.3, 29.0)	0.391
AST (U/L)	31.2 (18.8, 63.1)	33.4 (18.4, 498)	43.2 (22.7, 75.2)	37.0 (19.3, 50.4)	0.495
Creatinine (μmol/L)	70.5 (58.8, 95.0)	79.0 (62.0, 99.8)	97.0 (68.5, 121.5)	81.0 (59.0, 114.0)	0.133
Urea nitrogen (mmol/L)	6.3 (5.0, 7.0)	5.8 (4.3, 7.2)	6.0 (4.6, 7.5)	6.4 (5.1, 8.6)	0.512
Serum glucose (mmol/L)	7.3 (6.1, 8.8)	7.2 (6.2, 8.3)	7.3 (6.3, 8.8)	7.2 (6.3, 10.5)	0.994
WBC count (10*^9^/L)	11.6 (94, 14.4)	11.2 (9.5, 13.2)	11.9 (9.7, 15.4)	11.6 (8.8, 14.5)	0.300
Neutrophil count (10*^9^/L)	10.0 (7.1, 12.8)	9.5 (7.5, 12.0)	10.46 (8.6, 13.7)	10.24 (7.6, 11.9)	0.077
Lymphocyte count (10*^9^/L)	0.9 (0.6, 1.4)	0.9 (0.6, 1.4)	0.9 (0.6, 1.2)	0.8 (0.7, 1.2)	0.944
Monocyte count (10*^9^/L)	0.7 (0.5, 1.0)	0.7 (0.5, 0.9)	0.8 (0.6, 1.0)	0.7 (0.6, 1.1)	0.958
Serum potassium (mmol/L)	3.8 (3.4, 4.0)	4.0 (3.4, 4.0)	4.0 (3.3, 4.4)	4.0 (3.4, 4.1)	0.319
Serum chloride (mmol/L)	106.0 (103.0, 108.3)	101.0 (97.0, 103.0)	107.0 (104.0, 109.3)	104.0 (101.0, 106.0)	< 0.001
Serum calcium (mmol/L)	2.2 (2.1, 2.3)	2.2 (2.1, 2.3)	2.2 (2.0,2.3)	2.1 (2.0, 2.3)	0.055
AG (mmol/L)	14.1 (12.2, 16.5)	14.6 (12.0, 17.2)	16.8 (14.2, 19.4)	15.1 (12.6, 16.9)	0.003
Preoperative malperfusion (n, %)	7, 14.9	12, 21.8	13, 28.3	12, 34.3	0.190

Continuous variables that satisfy normal distribution are expressed by mean and standard deviation, and continuous variables that do not satisfy normal distribution are expressed by median and quartile. Categorical variables are expressed by number and percentage.

Group A, CO_2_CP ≥ 22.5 mmol/L + serum sodium >138.5 mmol/L; group B, CO_2_CP ≥ 22.5 mmol/L + serum sodium ≤ 138.5 mmol/L; group C, CO_2_CP < 22.5 mmol/L + serum sodium > 138.5 mmol/L; group D, CO_2_CP < 22.5 mmol/L + serum sodium ≤ 138.5 mmol/L; *CAD* coronary artery disease, *SBP* systolic blood pressure, *DBP* diastolic blood pressure, *ALT* alanine transaminase, *AST* aspartate transaminase, *WBC* white blood cell, *CO*_*2*_*CP* CO_2_ combing power, *AG* anion gap.

**Figure 2:**
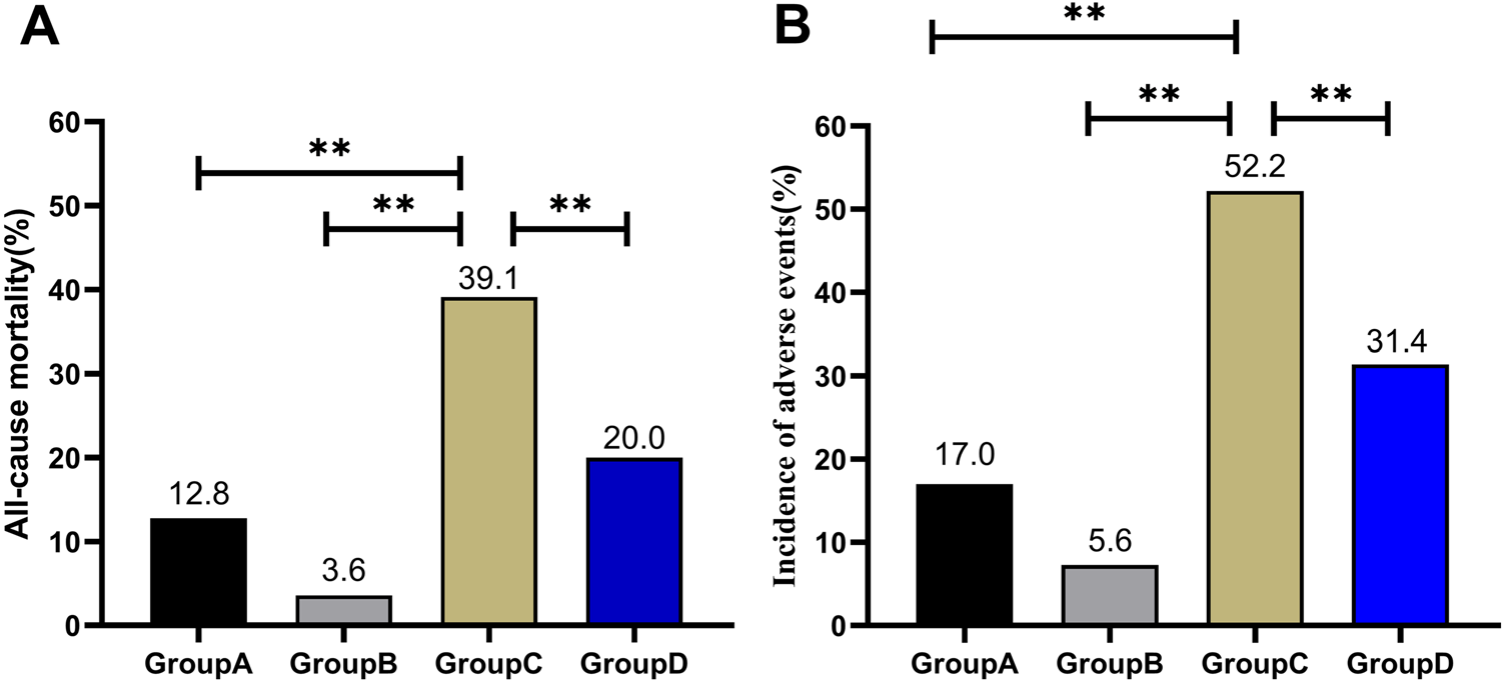
Comparison of observation endpoints according to CO_2_CP combined with serum sodium levels. (**A**) All-cause death; (**B**) adverse events.

The incidences of adverse events and mortality were significantly the highest in CO_2_CP < 22.5 mmol/L + serum sodium > 138.5 mmol/L group (all *P* < 0.01, Fig. 2A,B).

### Univariable and multivariable predictors of 30-day all-cause mortality in patients with ATAAD

The results of univariate and multivariate analysis using logistic regression analysis adjusting for other potential predictors of mortality were shown in Tables 4 and 5. Age, gender, serum glucose, preoperative malperfusion, CO_2_CP and sodium variables were incorporated into the multivariate logistic model. After adjusting for these covariates, results showed that CO_2_CP < 22.5 mmol/L combined with serum sodium > 138.5 mmol/L was more suitable for predicting the all-casue mortality of ATAAD patients (OR = 6.073, 95% CI 2.557–14.425, *P* < 0.001, Table 5).

**Table 4:** Independent predictors of mortality in univariate logistic regression analysis.

Variables	Univariate logistic regression analysis		
	OR	95% CI	*P*
Age (years)	1.010	0.977–1.044	0.561
Male (n, %)	0.865	0.385–1.945	0.726
Hypertension (n, %)	0.790	0.316–1.971	0.613
Diabetes (n, %)	0.400	0.035–4.554	0.460
CAD (n, %)	0.913	0.187–4.445	0.910
Smoking (n, %)	0.802	0.369–1.742	0.577
Drinking (n, %)	0.907	0.417–1.973	0.806
Heart rate (beats/min)	1.005	0.981–1.029	0.712
SBP (mmHg)	1.003	0.988–1.017	0.716
DBP (mmHg)	1.015	0.993–1.037	0.189
ALT (U/L)	1.000	0.998–1.002	0.822
AST (U/L)	1.000	0.998–1.001	0.720
Creatinine (μmol/L)	1.002	0.999–1.006	0.168
Urea nitrogen (mmol/L)	1.037	0.998–1.152	0.098
Serum glucose (mmol/L)	1.112	1.005–1.230	0.040
WBC count (10*^9^/L)	1.044	0.946–1.151	0.392
Neutrophil count (10*^9^/L)	1.031	0.935–1.136	0.544
Lymphocyte count (10*^9^/L)	0.918	0.489–1.729	0.792
Monocyte count (10*^9^/L)	1.227	0.437–3.449	0.697
Serum potassium (mmol/L)	1.107	0.615–1.993	0.735
Serum chloride (mmol/L)	1.017	0.965–1.072	0.521
Serum calcium (mmol/L)	0.173	0.024–1.241	0.081
Serum sodium (mmol/L)	1.115	1.039–1.198	0.003
CO_2_CP (mmol/L)	0.789	0.697–0.892	< 0.001
CO_2_CP < 21.5 mmol/L + serum sodium > 138.5 mmol/L	5.229	2.352–11.623	0.001
AG (mmol/L)	1.017	0.926–1.117	0.725
Preoperative malperfusion (n, %)	3.349	1.486–7.546	0.004

*CAD* coronary artery disease, *SBP* systolic blood pressure, *DBP* diastolic blood pressure, *ALT* alanine transaminase, *AST* aspartate transaminase, *WBC* white blood cell, *CO*_*2*_*CP* CO_2_ combing power, *AG* anion gap.

**Table 5:** Independent predictors of mortality in multivariate logistic regression analysis.

Variables	Multivariate logistic regression analysis		
	OR	95% CI	*P*
Age (years)	0.987	0.951–1.024	0.485
Male (n, %)	0.997	0.400–2.483	0.995
Serum glucose (mmol/L)	1.106	0.994–1.230	0.065
Preoperative malperfusion (n, %)	3.469	1.42–8.288	0.005
CO_2_CP < 22.5 mmol/L + serum sodium > 138.5 mmol/L	6.073	2.557–14.425	< 0.001

### Survival curve analysis

**Figure 3:**
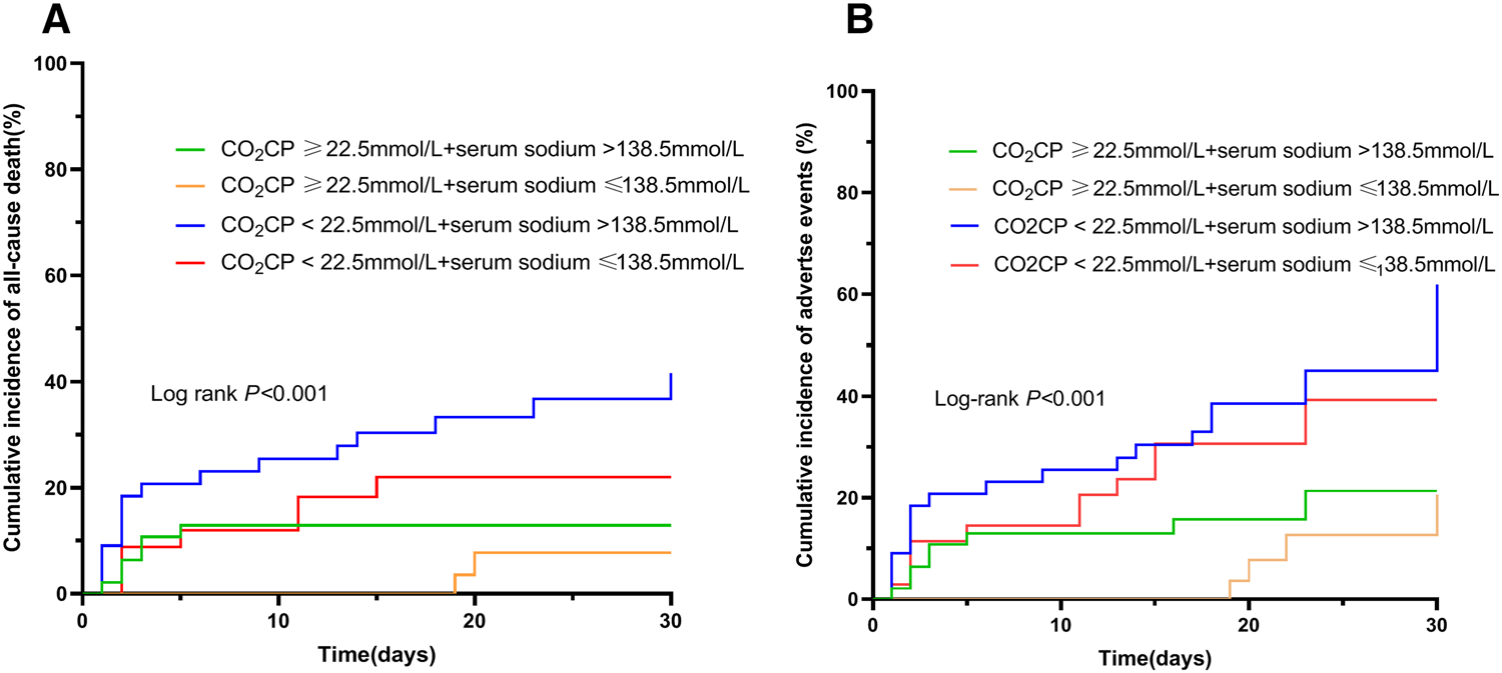
Kaplan–Meier survival curves for cumulative events in different CO_2_CP and serum sodium levels. (**A**) All-cause death; (**B**) adverse events.

Lastly, we conducted survival analysis of the combination of CO_2_CP and serum sodium levels of patients with ATAAD using the Kaplan–Meier curves to corroborate the above findings. As shown in Fig. 3A,B, Kaplan–Meier curves showed that combination low CO_2_CP levels with high serum sodium levels correlated with a higher incidence of all-cause deah and adverse events in ATAAD (log-rank *P* < 0.05).

## Discussion

In this study, we demonstrted that the admission levels of CO_2_CP combined with serum sodium may be a potential predictive marker for determining the postoperative all-cause mortality and adverse events in patients with ATAAD, this remained significant even after adjusting for confounders. The combination of CO_2_CP and serum sodium could improve the predictive efficiency of 30-day in-hospital mortality during hospitalization.

The actual mechanisms by which decreased CO_2_CP leads to adverse outcomes in patients with ATAAD remain unclear. Acidity is a well-characterized factor associated with initiation and development of cardiovascular diseases. Accumulative evidence indicates that lower pH levels in the extracellular space and the circulation promoted the progression of AAD^28^. Extracellular acidity is generated mainly by aortic vascular smooth muscle cells through production of proton and lactic acid^29^, which is maintained by a combination of oxygen depletion from malperfusion, increased intracellular H^+^ efflux and high activity of the carbonic anhydrases, CAIX and CAXII, which results in the acceleration of extracellular CO_2_ to HCO_3_^−^ and H^+^ in context of AAD-produced hypoxia. In turn, severe metabolic acidosis (a lower CO_2_CP in serum) not only damages the central nervous system but also induces ventricular arrhythmias and myocardial systolic disability. Moreover, acidosis could alter systemic vascular resistance promoting to aggravation of circulatory shock, leading to progressive cellular hypoxia and eventually causing end-organ failure. Patients with aortic dissection initially activate the coagulation fibrinolytic system^30^, accompanied by d-dimer and micro thrombosis in capillaries, causing abnormal blood flow rate, oxygen exchange obstacle, and glucose transformation into pyruvic acid under anaerobic conditions. The abnormally activated anaerobic glycolysis pathway mediates accumulation of pyruvic acid and lactic acid, ultimately exerting metabolic poisoning^31,32^. This acidic environment further drives the initiation and progression of complications as well as the poor prognosis. Severe metabolic acidosis not only damages the central nervous system but also induces ventricular arrhythmias and weakened myocardial systolic ability, which is fatal. Several studies verified the association of low HCO_3_^−^ levels with poor clinical outcomes, including haemodialysis and other treatment^33–35^. Chin Sang Ong et al.^36^ also found that severe acidosis was associated with higher postoperative mortality (OR = 13.9, *P* = 0.001) in 298 patients with ATAAD, suggesting that severe acidosis may be a strong predictor of postoperative mortality. In addition, CO_2_CP as metabolic indicator of acidosis and alkalosis was easier diagnoses than HCO_3_^−^ level in clinic, suggesting that evaluation of the acid–base homeostasis for patients with ATAAD in the emergency department would be accessible.

Hypoperfusion syndrome is a serious aortic dissection complication caused by the true and false lumen formation after aortic intima tear and the aortic branch vessel blockage by the torn inner membrane, leading to peripheral organ ischaemia and dysfunction before affecting early survival^37^. Czerny et al.^38^ revealed that 33.6% of patients were subjected to preoperative malperfusion, and the combined malperfusion syndrome was considered as an independent predictor of poor aortic dissection prognosis in a retrospective analysis of 2137 patients with ATAAD, which is similar to our study (OR = 3.335, 95% CI 1.430–7.777, *P* = 0.005).

The serological examination is considered as a suitable and simple way to assess a patient’s clinical status. Previous studies reported that serum sodium level change may affect vascular functions^39^. Experimental studies by Oberleithner^40^ revealed a 20% strengthened cell stiffness following the increased sodium concentration from 135 to 145 mmol/L and the decreased activity of endothelium-type nitric oxide synthase in endothelial cells, suggesting that alteration of serum sodium concentration also affect vascular endothelial function and vascular tone. In this study, we found that increased serum sodium was associated with in-hospital mortality in patients with ATAAD. More importantly, our data confirmed that the combination of CO_2_CP and serum sodium strengthened the predictive value for all-cause mortality in ATAAD, enhancing risk discrimination and providing important prognostic information for short-term follow-up after Bentall procedure.

An insight into the biomarker underlying complicated clinical scenarios is critical for developing primary or secondary preventions of ATAAD^9^, like as CO_2_CP and serum sodium. In agreement with previous studies, our results suggest that the hazardous effect of low CO_2_CP and high serum sodium appeared to be prominent in ATAAD patients with preoperative malperfusion, emphasising the requirement for intensive care and therapy. Combination of admission CO_2_CP and serum sodium, as the readily available parameters, easily simple for routine implementation in clinical practice, which can help clinicians choose more accurate therapies or early interventions for patients with ATAAD. Thus, we illustrates such a scenario that ATAAD patients with low CO_2_CP level and high serum sodium level at admission should be given more attention, and closely monitored hemodynamic indexes and malperfusion.

Overall, the present study highlights the predictive value of preoperative CO_2_CP combined with serum sodium in identifying patients with ATAAD who were at high risk of 30-day mortality and adverse events, better than that of a single elevated serum sodium and a single diminished CO_2_CP. Combination of CO_2_CP and serum sodium might be a better application in ATAAD managment.

### Study limitations

Our study remains several limitations. First, based on a single-centre study with a small sample size, the conclusion will be further verified by a multicenter study. Second, the combination of serum HCO_3_^−^, base excess and pH value could be a better supplement. Third, the predictive CO_2_CP combined with serum sodium for the long-term prognosis in patients with ATAAD should be surveyed. Overall, further investigation of the underlying mechanism is supposed to the multiple-centre, large and prospective studies.

## Conclusion

Combination of admission CO_2_CP and serum sodium levels presented a better predictive value for 30-day all-cause mortality and adverse outcomes of in-hospital patients undergoing Bentall procedure for ATAAD.

## Data Availability

The datasets generated and analyzed during the current study are available from the corresponding author on reasonable request.
